# Recording Neural Reward Signals in a Naturalistic Operant Task Using Mobile-EEG and Augmented Reality

**DOI:** 10.1523/ENEURO.0372-23.2024

**Published:** 2024-08-07

**Authors:** Jaleesa S. Stringfellow, Omer Liran, Mei-Heng Lin, Travis E. Baker

**Affiliations:** ^1^Center for Molecular and Behavioral Neuroscience, Rutgers University, Newark, New Jersey 07102; ^2^Department of Psychiatry & Behavioral Neurosciences, Cedars-Sinai Virtual Medicine, Los Angeles, California 90048

**Keywords:** anterior cingulate cortex, augmented reality, EEG, real-world neuroscience, reinforcement learning, reward

## Abstract

The electrophysiological response to rewards recorded during laboratory tasks has been well documented, yet little is known about the neural response patterns in a more naturalistic setting. Here, we combined a mobile-EEG system with an augmented reality headset to record event-related brain potentials (ERPs) while participants engaged in a naturalistic operant task to find rewards. Twenty-five participants were asked to navigate toward a west or east goal location marked by floating orbs, and once participants reached the goal location, the orb would then signify a reward (5 cents) or no-reward (0 cents) outcome. Following the outcome, participants returned to a start location marked by floating purple rings, and once standing in the middle, a 3 s counter signaled the next trial, for a total of 200 trials. Consistent with previous research, reward feedback evoked the reward positivity, an ERP component believed to index the sensitivity of the anterior cingulate cortex to reward prediction error signals. The reward positivity peaked ∼230 ms with a maximal at channel FCz (M = −0.695 μV, ±0.23) and was significantly different than zero (*p* < 0.01). Participants took ∼3.38 s to reach the goal location and exhibited a general lose-shift (68.3% ±3.5) response strategy and posterror slowing. Overall, these novel findings provide support for the idea that combining mobile-EEG with augmented reality technology is a feasible solution to enhance the ecological validity of human electrophysiological studies of goal-directed behavior and a step toward a new era of human cognitive neuroscience research that blurs the line between laboratory and reality.

## Significant Statement

Building on decades of experimental, computational, and theoretical analyses of reinforcement learning in animal and humans, the present study reveals for the first time that scalp-recorded electrophysiological signals associated with the anterior cingulate cortex sensitivity to reward prediction error signals is dynamically modulated by rewards in humans freely navigating a more realistic environment and that participants performed the task in accordance with reinforcement learning theory.

## Introduction

The ability to utilize reward information to adaptively guide behavior to meet current and future goals is essential to successfully navigate through a busy day. Extensive theoretical and empirical work based on simplistic laboratory tasks indicate that goal-directed behavior is largely mediated by key neural targets of the mesocorticolimbic reward system [e.g., orbitofrontal cortex, striatum, prefrontal cortex and anterior midcingulate cortex (ACC)] and a dopaminergic teaching signal tethered to prediction of reward outcomes during trial-and-error learning (i.e., reward predication error signals, RPEs), as indicated by animal (Schultz, 1998; Sutton and Barto, 1998) and translational human research (Haber and Knutson, 2010). Current thinking holds that phasic bursts and dips in dopamine activity are elicited when events are, respectively, “better than expected” (positive RPE) and “worse than expected” (negative RPE; Schultz, 2011). RPEs allow the mesocorticolimbic reward system to learn to detect rewards, predict future rewards, and use reward information to select and motivate behavior toward a goal (Niv et al., 2005; Garrison et al., 2013). Although the neural circuit involved in RPE-related processes has been well defined in simplified and controlled experimental settings, it is unclear how accurately these processes translate to more complex, naturalistic situations.

To address this issue, we tested a novel mobile-EEG and augmented reality (AR) paradigm aimed to record RPE-related neural activity during realistic goal-directed behavior. We focused on the role of the ACC in goal-directed behavior and the application of AR to achieve ecological validity in an experimental setting. A prevailing hypothesis holds that the ACC utilizes RPEs to learn the value of rewards for the purpose of selecting and motivating the execution of goal-directed behavior (Holroyd and Coles, 2002; Holroyd and Yeung, 2012). In humans, the reward function of ACC can be investigated using an event-related brain potential (ERP) called the reward positivity (Baker and Holroyd, 2011; Sambrook and Goslin, 2015). The reward positivity is observed as a differential response in the ERP to positive and negative feedback received during choice tasks and is believed that the impact of positive and negative RPEs on the ACC modulates the amplitude of the reward positivity (Holroyd and Coles, 2002; Holroyd and Yeung, 2012). Converging evidence across multiple methodologies indicate that the reward positivity reflects an RPE signal and is generated by the ACC (Holroyd and Umemoto, 2016). While the reward positivity has been studied for decades in tasks requiring subjects to press buttons to make choices between options that pay out probabilistic rewards, these oversimplified tasks may fail to engage naturalistic cognitive processes.

Recent trends in neuroscience research are gravitating toward experiments that emulate naturalistic settings, leading to novel insights into cognition (Sonkusare et al., 2019). Cognition is now understood to be a dynamic, interconnected phenomenon, rather than a series of static and isolated operations. While naturalistic paradigms may not pinpoint the precise neural activity that can be revealed through highly controlled laboratory experiments (Rust and Movshon, 2005), incorporating elements of the real world, such as authentic stimuli or behaviors, may possibly reveal unobserved dimensions of brain processes that have previously been examined under standard laboratory conditions. For example, locomotion, an understudied component in human cognitive research, is now recognized as a critical element that influences cognitive functions (Gramann et al., 2014; Stangl et al., 2023). The current study seeks to extend these findings by integrating augmented reality with mobile-EEG to allow participants to perform a reward task during free motion and by doing so, reveal reward-related processes in a setting that closely mirrors natural experiences.

**Figure 1.**
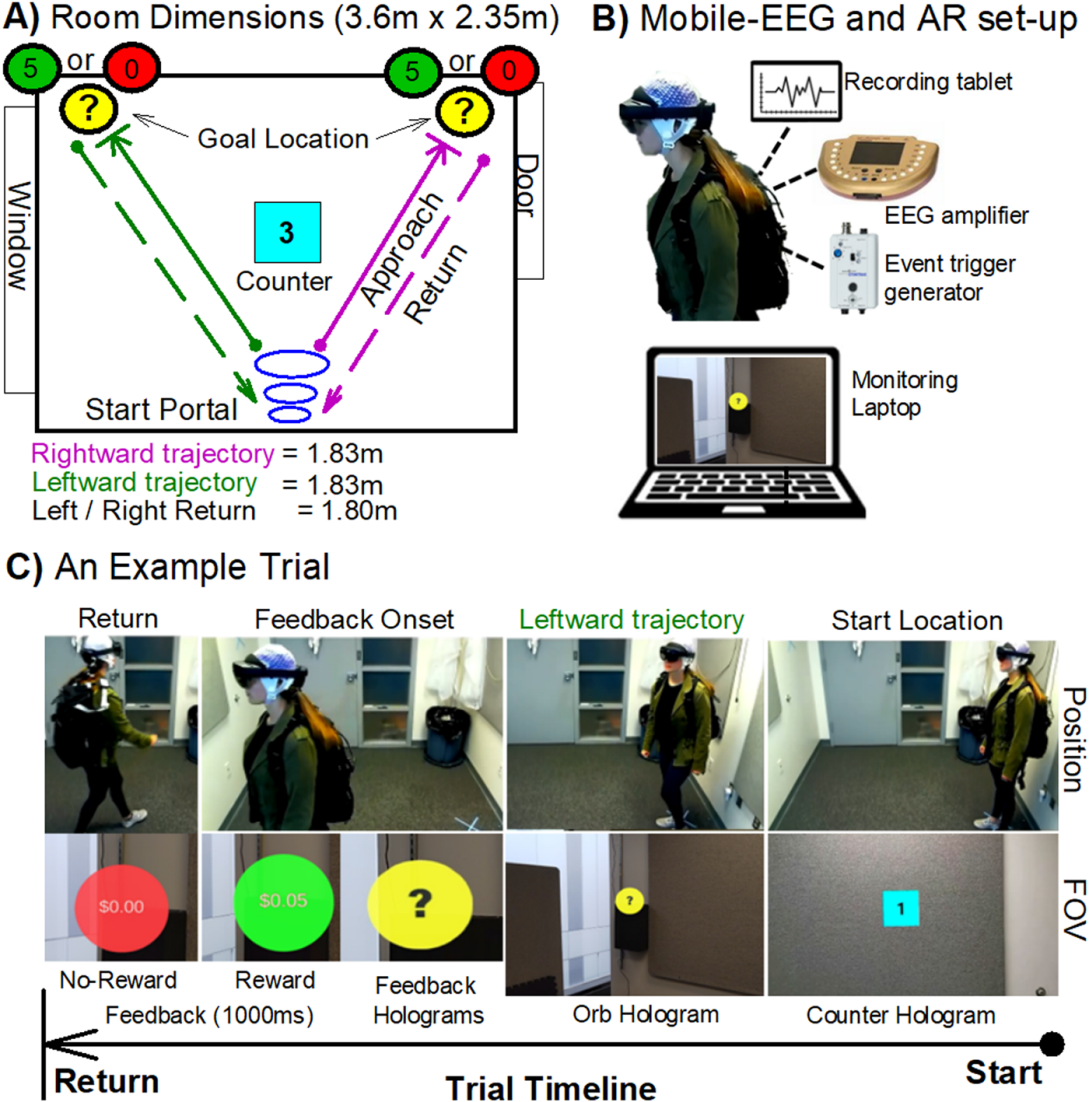
Experimental setup. Mobile-EEG and AR operant chamber paradigm. ***A***, Dimensions of the physical room and placement of the task holograms. Purple and green lines denote rightward and leftward trajectories, respectively. ***B***, AR hardware and EEG setup, which include a HoloLens 2 (Microsoft), V-amp EEG system with 16-channel BrainVision actiCAP electrodes, and StimTrak system to record event triggers (audio; Brain Products), a tablet to record EEG data and a standard laptop to monitor subjects’ field of view (FOV) and to provide instructions. ***C***, An example of a rightward trajectory in the AR task (see Extended Data Fig. 1-1 for a video that depicts trial-to-trial sequence of events).

Here, we seek to move beyond simplistic lab-based experiments by measuring RPE-related neural activity and behavior in humans while they freely performed a two-choice operant task in a naturalistic environment. Here, we leveraged technological advances in mobile-EEG and head-mounted AR glasses to investigate RPE-related processes in humans freely navigating a room to find rewards. AR is an interactive experience where virtual objects are overlayed onto real-world objects by computer-generated perceptual information across multiple sensory modalities (e.g., visual, auditory) using a special kind of optic glasses (Fig. 1*B*; HoloLens 2, Microsoft). AR is seamlessly interwoven with the physical world such that it is perceived as an immersive aspect of the real environment. In this way, AR alters one's ongoing perception of a natural environment and can therefore be an ideal solution for providing experimental control of stimulus in any realistic setting (Krugliak and Clarke, 2021).

To record the reward positivity in a naturalistic operant paradigm, we designed an AR task using the HoloLens 2 in which subjects began each trial in a start location marked by three-dimensional floating purple rings, and once a 3 s floating timer ended, subjects were instructed to walk toward one of two floating yellow orbs in the west and east side of the room. Once subjects were within half a meter and fixated on the floating orb, the orb would change into either a reward cue or no-reward cue. It is at the onset of the reward cue we measured the reward positivity. Following each feedback presentation, subjects walked back to the start location and started the next trial. Because the reward positivity has yet to be investigated in a naturalistic setting, we examined whether the reward cues interwoven within the physical world could elicit this ERP component. In sum, we propose that the melding of mobile-EEG and AR provides a unique opportunity to study the ecological validity of RPE-related neural signals evoked during goal-directed behavior as it holds promise for integrating experimental, computational, and theoretical analyses of laboratory behavior in a natural environment (Krugliak and Clarke, 2021).

## Materials and Methods

### Participants

Twenty-five adults (24 right-handed, 6 male and 19 female, aged 18–43 years old [*M* = 23, SE = 6]) were recruited from the Newark community and Rutgers University Department of Psychology participant pool using the SONA system. The study adhered to the principles expressed in the 1964 Declaration of Helsinki. Informed consent was obtained from all participants. Each participant received either course credit or $25 an hour plus $5 task bonus for their participation. Before the experiment, participants were screened for neurological symptoms and histories of neurological injuries (e.g., head trauma) and then asked to fill out the Edinburgh Handedness Inventory (Oldfield, 1971). After the experiment, participants filled out the Everyday Spatial Questionnaire. All participants provided written consent before the experiment. Of the 25 participants, data from four participants were excluded due to excessive artifacts in the EEG data (*n* = 1) and technical issues during recording (*n* = 3).

### AR operant task

Since the operant task has been the gold standard laboratory task for studying reinforcement learning and goal-directed behavior in freely moving animals (Eagle and Robbins, 2003; Schifani et al., 2017), we utilized mobile-EEG and AR technology to create a full-scale human version of an operant chamber in order to implement a two-alternative forced choice task with ambiguous goal location cues and feedback stimulus (Fig. 1*A*). The AR operant chamber was enclosed inside the lab's physical space of a 2.13 m-by-2.13 m room and was constructed using commercially available computer software (Unity version 2019.2; https://unity.com). The AR environment was provided through Microsoft HoloLens 2 (HL2) head-mounted holographic system, which tracked participants’ head positions and eye gaze fixation during task performance. To note, the 3D virtual objects or “holograms” that HoloLens renders appear in the holographic frame directly in front of users’ eyes. Holograms add light to the physical environment, which means that subjects see both the light from the display and the light from the surrounding environment. While “3D virtual object” may be the proper term for AR space, the Microsoft HL2 uses the term “hologram” and “holographic representations” in all its documentation, programing, and hardware in accordance with its mixed reality application and design. Thus, for the purpose of simplicity, we will refer to the 3D virtual objects as holographic images. Continuous EEG was recorded from 16 actiCAP slim electrodes using a mobile V-Amp amplifier system (Brain Products; Fig. 1*B*).

Prior to the experiment, participants were trained to use the HL2 system and performed the eye calibration setup to ensure accurate eye tracking. All participants were queried verbally about their familiarity with AR using the HL2 headset. While it is highly likely that participants have in fact interacted with AR before (e.g., social media filters) using smartphone or tablets, no one reported previous use of AR with the HL2 headset. Each participant underwent a 5 min tutorial using the HL2 (HoloLens Tips App). This application served as an instructional guide, demonstrated how to navigate within an augmented reality space, manipulate holograms, and interact with the main menu and other applications specific to the HL2 system. Following tutorial and eye calibration, participants were instructed to set up the experiment, while the experimenter tracked their point-of-view (POV) remotely on a tablet. The setup consisted of placing three holograms within the room, the start portal (ascending blue rings) and two yellow floating orbs marking left and right goal locations. To ensure consistent start and target locations for each participant, subjects were instructed to position the start portal over a designated floor sticker. Additionally, they placed two yellow orbs against a left and right goal box on the back wall. The yellow orbs automatically adjusted to the participants’ eye level. For a detailed visual representation of the trial-to-trial sequence of events, please see extended data video supporting Figure 1 labeled as Extended Data Figure 1-1.

10.1523/ENEURO.0372-23.2024.video1 Movie 1 Download Movie 1, MP4 file.

Once the holograms were placed in the correct locations, participants were instructed to stand in the start portal and to press a virtual button to begin the experiment. Once standing in the start location, a holographic countdown timer would appear for 3 s, and participants were instructed to make their choice at the end of the countdown. Depending on their choice, participants could move toward the left or right goal location (yellow floating orb), and once standing in front of the orb and looking at it as detected using eye tracking, the floating yellow orb turned either green to signify a reward (5 cents) or red signifying no-reward (0 cents; Fig. 1*C*). To be more specific, there was no delay between the person standing in front of the orb and detecting that their eyes are fixated on it. The eye tracking is per frame, so the system checks on the same frame that they are within range. If they were not looking at the orb when they were in position, the app would wait. In particular, there are two conditions that need to be met: (1) distance from orb (<0.5 m) and (2) eyes on orb. As long as those two conditions are met on the same frame, the reward gets revealed. Since we are at 90 frames per second, the wait is never longer than 11 ms. Furthermore, despite not inquiring about colorblindness among participants, the study's design incorporated both color and textual information in the feedback. In the reward condition, targets were marked green and explicitly displayed a reward value of 5 cents, whereas in the no-reward condition, targets were red and indicated a zero cents loss. Consequently, this approach ensured that participants, irrespective of color vision deficiencies, could discern trial outcomes based on the visually presented monetary value.

Following the feedback (duration 1,000 ms), participants then walked back to the start location to begin the next trial. The task consisted of four blocks (50 trials per block), separated by self-timed rest breaks that presented their cumulative earnings. Unknown to them, on each trial the type of feedback was selected at random (50% probability for each feedback type), a necessity to record the reward positivity using a difference wave approach (Cockburn and Holroyd, 2018). At the end of the experiment, participants were informed about the probabilities and were given a $5 performance bonus. Before beginning the task, participants were informed that the rewards accrued in this task directly corresponded to actual cash to be received upon the conclusion of the study. To maintain transparency and engagement, the accumulated earnings were displayed to the participants during the rest period at the end of each block.

### HL2 data acquisition

The software running on HL2 recorded and exported experimental and behavioral data for each participant (Fig. 1*A*). The sampling rate of the HL2 was 60 Hz and recorded via Bluetooth to a comma-separated values file on a remote computer in the adjacent room. Time-locked EEG markers were sent to the EEG system by converting an 11 ms event-related audio signal or sine wave (e.g., countdown onset and offset, feedback onset and offset) to a TTL pulse using the BrainVision (BV) StimTrak system (Fig. 1*B*). To note, there was a delay (<200 ms) between visual and auditory onset which could not be corrected in the HL2 programming platform, which resulted in a delay of the event triggers recorded in the EEG system. To correct for the output–input delay, the HL2 event markers and EEG triggers were synchronized using custom-written MATLAB scripts that added 200 ms to each trial. HL2 activity was monitored and controlled using both the web browser access (Microsoft Device Portal) and the HoloLens Application (Microsoft Corporation, version 1.1.70) on an external Microsoft tablet and laptop.

### Electrophysiological data recording

The electroencephalogram (EEG) was collected using a 16-channel actiCAP snap system (Brain Products) with 12 scalp electrode sites (C3, C4, Cz, F3, F4, FC1, FC2, FCz, P3, P4, P7, and P8) and four external electrodes. The EEG signals were referenced online to channel Pz with a ground at AFz, amplified using a portable V-Amplifier (max 16 electrodes), and recorded using BrainVision Recorder software (Brain Products). The slim electrodes sit ∼6 mm above the scalp, and due to the HoloLens hardware, the brow pad and brow pad foam needs to fit pretty tightly against the forehead, so it does not move and thus we were not able to use the frontal channels (e.g., Fpz, Fz). To ensure that the subject was comfortable while wearing the device and walking, we restricted our frontal channels to Fp2 (and a VEOG channel placed below the eye) to detect vertical eye activity (Blinks), and a left and right horizontal channel was used to detect horizontal eye activity (saccades). Electrode impedances were maintained below 20 kΩ. The sampling rate was set to 1,000 Hz. The electrooculogram (EOG) was recorded for the purpose of eye artifact correction. Horizontal EOG was recorded from the external canthi of both eyes, and vertical EOG was recorded from the suborbital and infraorbital regions of the right eye. To note, by convention mastoid sites (M1 and M2) are collected to rereference offline. However, these electrodes were removed from the dataset due to excessive noise and were not used in the analysis (Lin et al., 2022). To note, continuous EEG was recorded with a mobile V-Amp amplifier from 16 actiCAP slim electrodes (C3, C4, Cz, F3, F4, FC1, FC2, FCz, P3, P4, P7, and P8).

### Electrophysiological data analysis

EEG data were analyzed offline using BrainVision Analyzer 2 (Brain Products). The EEG signals were filtered using a fourth-order digital Butterworth filter with a bandpass of 0.1–20 Hz. Eye artifacts were corrected using independent component analysis (ICA) method with a mean slope algorithm for blink detection and infomax-restricted algorithm used for ocular artifact correction (Jung et al., 2000). The EEG data were then segmented into 1,000 ms epochs spanning from −200 to 800 ms from feedback onset. The segmented data was then baseline corrected using a mean voltage range from −200 to 0 ms. The data was then rereferenced using an average reference created from the following channels: C3, C4, Cz, F3, F4, FC1, FC2, FCz, P3, P4, P7, and P8. Segments containing muscular and other artifacts were removed using the following criteria: (1) a maximal voltage step of 35 μV/ms, (2) a maximal difference of values in intervals of 150 μV, and (3) lowest allowed activity values in intervals of 0.5 μV. Following artifact rejection, channels containing artifacts that exceed 5% of the data were identified and interpolated using Hjorth-nearest neighbor algorithm (Hjorth, 1975). In the process of artifact rejection, the 5% criterion represents a commonly used yet arbitrary threshold for each channel, determined by the percentage of noise present in the data. In this study, only one participant exhibited a channel (C4) that required interpolation, employing the Hjorth algorithm, with a noise level of 1.042% across four segments. Prior to averaging, we corrected the latency jitter in the ERP across trials by applying the Adaptive Woody Filter method (AWF) using a 100–300 ms time window with 50 ms step interval at channel FCz (Woody, 2006; Li et al., 2009; Gavin et al., 2019; Lin and Baker, 2022).

ERPs were created for each participant and electrode by averaging the single-trial EEG data according to feedback type (reward and no-reward feedback). The reward positivity was then evaluated as a difference wave by subtracting reward from no-reward ERPs. The size of the reward positivity was then determined by identifying the peak amplitude of the difference between the reward and no-reward ERPs within a 100–400 ms window after feedback onset. The difference wave method was recommended in a meta-analysis and isolates the reward positivity from other ERP components (Sambrook and Goslin, 2015). Local maxima peak detection was used on the difference wave to extract peak amplitude of the reward positivity at each channel within the 100–400 ms time window (Baker et al., 2016a,b, 2020; Biernacki et al., 2020). Peak amplitudes, at each channel, were also tested against zero using one-sample *t* test with a significance level of *a* = 0.01 to confirm the presence of the reward positivity.

### Behavioral analysis

Operant task performance measures included the following: (1) reaction time (RT) reported in seconds and measured from countdown offset to feedback onset (start RT) and from feedback location to the start location (return RT); (2) postfeedback RT measured from feedback onset back to start location; and (3) win-stay and lose-shift behavior, defined by choosing the same location (right or left) after a reward feedback and selecting the alternative location after no reward feedback, respectively. We excluded trials with RTs slower than 5% of the higher boundary. Postfeedback performance was analyzed using general linear models with feedback (win/loss), behavior (stay/shift), and task by the first or second half of the experiment (i.e., total number of trials recorded and then divided by 2) as within-subject factors, followed by post hoc tests with a significance level of *α* = 0.05. To note, while each participant was required to complete 200 trials, technical issues resulted in incomplete recording of all 200 trial markers for three subjects (*n* = 168; *n* = 150; and *n* = 150). For the purpose of clarity, we use the term choice behavior when referring to trial-to-trial behavior (e.g., select left or right goal location) and postfeedback choices (e.g., win-stay, lose-shift) when referring to the participant's subsequent action following feedback, which involves either maintaining their initial choice (staying) or altering it (switching; Baker et al., 2020).

## Results

### Task performance

**Figure 2.**
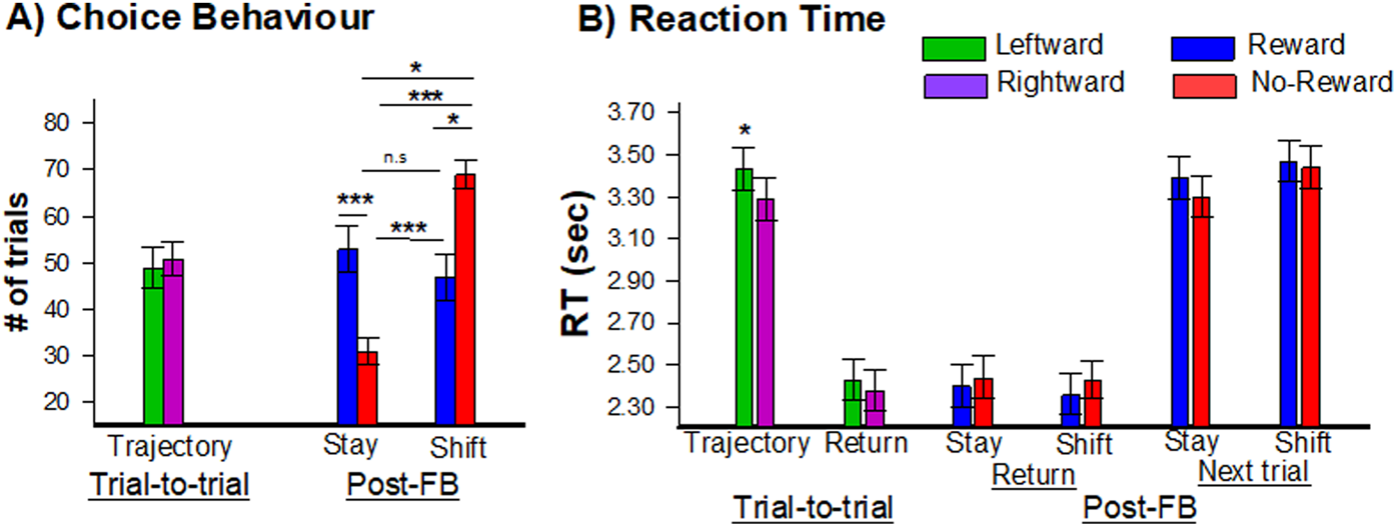
Results of the AR operant chamber performance analysis. Behavioral analysis for choice (***A***) and reaction time (***B***). Green and purple bars denote leftward and rightward trajectories, and blue (reward) and red (no-reward) bars denote postfeedback return behavior (feedback location to start location) and next trial behavior (start location to feedback). To note, although not shown here, reaction time was slower during the return to start location following no-reward cues (*M* = 2.43 s ± 0.10 s) compared with reward cues (*M* = 2.38 s ± 0.11 s), *p* < 0.05. Significant effects are shown as follows: *p < 0.05, **p < 0.01, ***p < 0.001 (two-tailed). Error bars denote standard error.

On average, participants completed 195 trials (standard deviation = 14, range = 150–200) and required ∼3.38 s (±0.93 s) to reach the feedback location, located roughly 2.49 m away. A two-way repeated-measures ANOVA on start-RT with Half (Half-1, Half-2) and Direction (Left vs Right) as factors revealed a main effect of Half, *F*_(1,21)_ = 6.35, *p* < 0.05, *η*^2^ = 0.23. Post hoc tests indicated faster RT for the second half (*M* = 2.39 s ± 0.10 s) compared with the first half of the experiment (*M* = 3.40 s ± 0.11 s), *t*_(21)_ = 8.87, *p* < 0.001, Cohen's *d* = 1.86 (Fig. 2*B*). Further, there was a main effect of direction, *F*_(1,21)_ = 4.33, *p* < 0.05, *η*^2^ = 0.17, showing that the participant approached the right target (*M* = 3.29 s ± 0.10 s) slightly faster than the left target (*M* = 3.43 s ± 0.10 s), *t*_(21)_ = −1.95, *p* = 0.06, Cohen's *d* = 0.42. Regarding choice behavior, no main effects or interactions were found.

Next, a repeated-measures ANOVA on postfeedback choice with feedback (win vs loss), behavior (stay vs shift), and task-half (Half 1 vs Half 2) as within-subject factors revealed a main effect of behavior *F*_(1,21)_ = 4.31, *p* < 0.05, *η*^2^ = 0.17. Post hoc analysis indicated that participants shifted (58% ±3.8) more often than stayed (42% ±3.8), *t*_(21)_ = 2.08, *p* < 0.05, Cohen's *d* = 0.81. This analysis also revealed an interaction between feedback and behavior, *F*_(1,21)_ = 15.38, *p* < 0.001, *η*^2^ = 0.42. Post hoc test indicated that participants shifted their responses more often following negative feedback (*M* = 68.3% SEM = ±3.5) compared with positive feedback (*M* = 47.1%, SEM = ±5.7), *t*_(21)_ = 3.67, *p* < 0.01, Cohen's *d* = 0.95 (Fig. 2*A*). In contrast, participants stayed with their response more often following positive feedback (*M* = 52.8%, ±5.7) compared with negative feedback (*M* = 31.7%, ±3.5), *t*_(21)_ = 3.67, *p* < 0.01, Cohen's *d* = 0.95 (Fig. 2*A*). To note, no differences were observed between win-stay and win-shift performance (*p* > 0.05), but a difference was observed between lose-shift and lose-stay (*p* < 0.001). Even though feedback was randomized, these results indicated that feedback influenced the participants’ subsequent behavior in this task. In regard to postfeedback RT, there was a main effect of Half, *F*_(1,21)_ = 4.23, *p* < 0.05, *η*^2^ = 0.18, indicating a faster RT for the second half (*M* = 2.39 s ± 0.11 s) compared with the first half (*M* = 3.45 s ± 0.10 s). Finally, a repeated-measures ANOVA on postfeedback RT during the return stage of the trial with feedback (win vs loss) and behavior (stay vs shift) as within-subject factors revealed a significant main effect of feedback, *F*_(1,21)_ = 4.02, *p* < 0.05, *η*^2^ = 0.16, indicating that RT was slower following no-reward cues (*M* = 2.43 s ± 0.10 s) compared with reward cues (*M* = 2.38 s ± 0.11 s), *t*_(21)_ = −2.01, *p* < 0.05, Cohen's *d* = 0.42 (Fig. 2*B*). No other main effects or interactions were observed.

### Reward positivity

**Figure 3.**
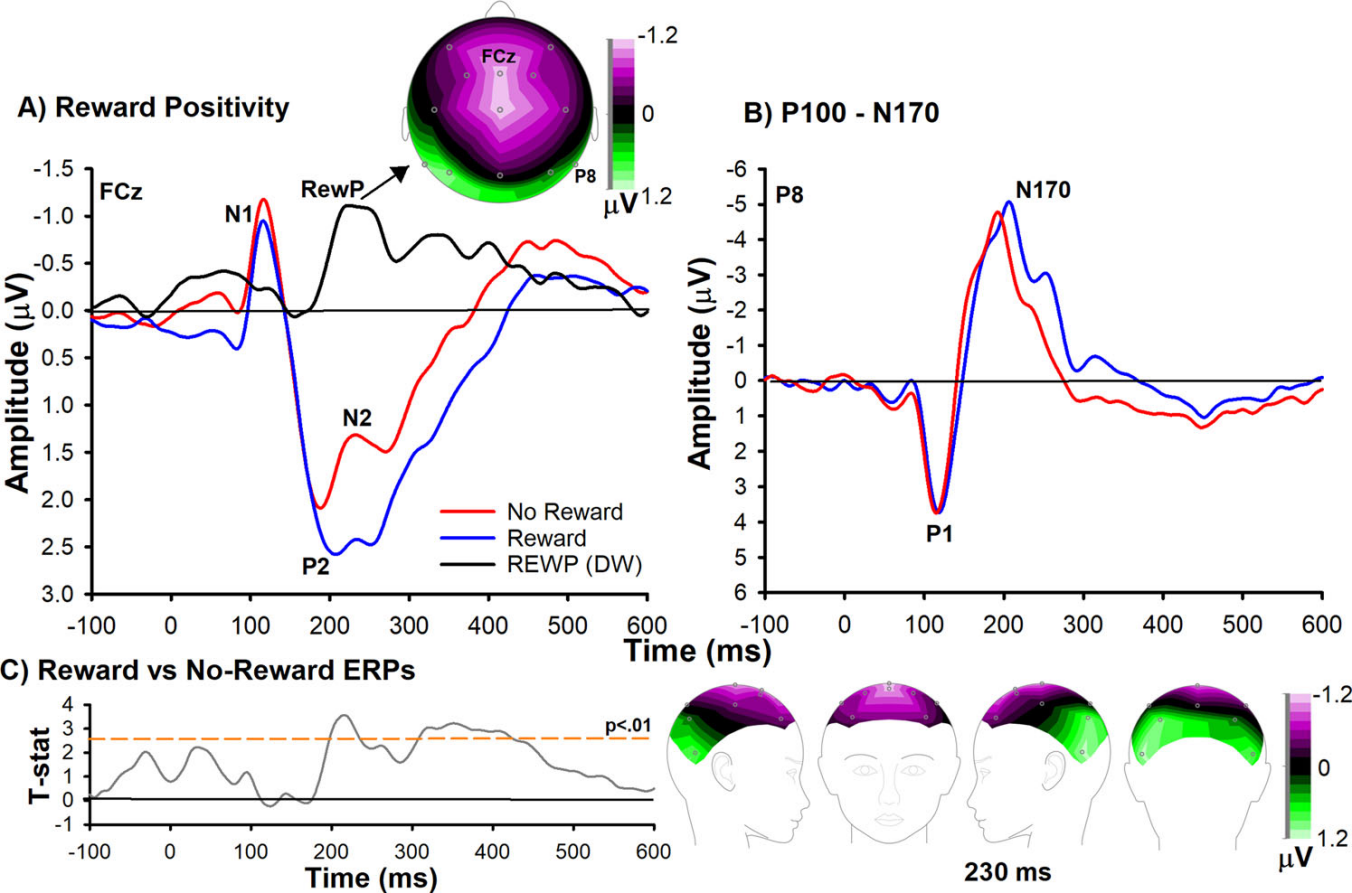
Reward positivity results. ***A***, ERPs elicited by reward feedback (blue), no-reward feedback (red), and a difference wave representing reward positivity (REWP, black) averaged across all blocks. Topoplots denote the amplitude of the reward positivity at 230 ms (top left and bottom right panel). ***B***, For the purpose of comparison, we plotted the ERPs over posterior channel P8 to highlight that the feedback stimulus presented in the AR paradigm is capable of eliciting ERPs commonly associated with perceptual processing of the stimulus (N100 and N170). Data are associated with channel FCz (left panel) and P8 (right panel) and negative is plotted up by convention. ***C***, For illustrative purposes, we show the *t* statistic across time between reward and no-reward ERPs averaged across frontocentral scalp locations FCz and Cz. Dashed lines denote the significant threshold (*p* < 0.01). Note the time regions that exceeded this threshold correspond to the time range of the reward positivity.

Because the reward positivity to holographic feedback stimuli has not yet been investigated, we examined whether holographic rewards encountered during free navigation elicit this ERP component. Figure 3 presents stimulus-locked grand averages for both feedback conditions at channel FCz. Consistent with previous research, the reward positivity elicited by monetary rewards was clearly evident in the difference wave (*M* = −0.69 μV; SEM = ±0.23 μV) peaking 230 ms after feedback onset (Fig. 3*A*, solid lines) and was significantly different from zero, *t*_(20)_ = −2.95, *p* < 0.01, Cohen's *d* = −0.62). Further, as shown in Figure 3*A*, the frontocentral distribution is consistent with the identification of this ERP component as the reward positivity (Miltner et al., 1997), indicating that holographic-related feedback can elicit the reward positivity in freely moving participants. These results confirm that the AR operant chamber paradigm elicited the reward positivity component. To note, this paradigm also elicited other common feedback-related ERP components, particularly the N100, P100, N170, and P200 (Fig. 3*B*), indicating that the AR task is capable of eliciting a broad spectrum of cognitive processes, including those related to perceptual processing, cognitive control, and contextual updating (Luck, 2014).

## Discussion

The electrophysiological response to rewards has been well documented during laboratory-based tasks, yet little is known about these responses in a more naturalistic environment. To examine this issue, we designed a novel AR operant task to test whether holographic reward cues presented in a naturalistic setting can elicit the reward positivity, an ERP component associated with ACC sensitivity to RPE signals. Our novel findings show that the reward positivity can be accurately recorded during naturalistic behavior, and participants performed the task in accordance with reinforcement learning theory. Foremost, the reward positivity peaked at ∼230 ms postfeedback with a frontocentral negative topographic distribution, replicating previous reward positivity studies using tasks displayed on desktop computers (Sambrook and Goslin, 2015). An influential theory of ACC function proposes that the ACC utilizes dopaminergic RPEs signals to learn the value of rewards for the purpose of selecting and motivating the most appropriate action plan directed toward goals (Holroyd and Coles, 2002; Holroyd and Yeung, 2012; Holroyd and Umemoto, 2016). Accordingly, it is believed that the impact of positive RPEs on the ACC following goal-directed feedback modulates the amplitude of the reward positivity (Holroyd et al., 2008; Baker and Holroyd, 2011; Holroyd and Umemoto, 2016). Converging evidence across multiple methodologies indicate that the reward positivity reflects an RPE signal and is generated by ACC (Holroyd and Yeung, 2012; Sambrook and Goslin, 2015; Holroyd and Umemoto, 2016). In particular, genetic, pharmacological, and neuropsychological evidence implicates dopamine in reward positivity production (Baker et al., 2016a); and source localization studies, simultaneous recording of EEG/fMRI data, and intracranial recording studies in rodents (Warren et al., 2015), nonhuman primates (Emeric et al., 2008), and humans (Ramakrishnan et al., 2019) indicate that the reward positivity is generated in the ACC. Together, these results indicated that the operant chamber AR task is capable of eliciting RPE-related ACC activity.

At a behavioral level, participants exhibited a lose-switch strategy and walked slower from the goal location to the start location following no-reward feedback, evidence that the AR task can drive adaptive learning. More specifically, participants shifted more often following negative feedback compared with positive feedback and repeated their response more often following positive feedback compared with negative feedback. These results indicate that feedback did in fact influence behavior and appears consistent with Thorndike's law of effect: if an action is followed by a reward or punishment, then that action will be more or less likely, respectively, to reoccur (Catania, 1999). Further, given that RPE signals are used for the purpose of action selection, these results could reflect the degree in which negative and positive RPEs modified trial-to-trial behavior. Further, we observed posterror slowing in walking speed following negative feedback. Posterror slowing, commonly measures in button press tasks, represents the amount that responses slowed on a trial following an erroneous behavioral response (or negative feedback) compared with a correct response (or positive feedback; Heydari and Holroyd, 2016; Schroder et al., 2020). Varying accounts suggest that posterror slowing is reflective of the degree RPE signal is utilized for future behavioral adaption (immediate reaction time slowing following errors), fitting with the proposed function of the prefrontal cortex and ACC in cognitive control and reinforcement learning (Yeung et al., 2004; Dutilh et al., 2012). While this result requires replication, it is worth noting that this is the first time posterror slowing has been observed beyond button press tasks.

Further, it is worth noting that participants exhibited an equal propensity to modify their response after a reward, demonstrating no difference between win-stay and win-shift performance. This observation deviates from heuristic learning models typically employed in various fields, including psychology, game theory, statistics, economics, and machine learning, where the win-stay strategy often dominates. However, this is the second time we replicated this result, previously reported in a study where participants navigated an immersive virtual reality T-maze task to locate rewards (Lin et al., 2022). Conversely, in traditional experiments where subjects are pressing buttons to make decisions on a computer screen, a higher proportion of win-stay responses compared with win-shift responses is observed (Baker et al., 2016b, 2020). One possible explanation for this discrepancy might lie in the differential cognitive demands between active navigation and simple button press tasks (Coddington and Dudman, 2019). During active navigation, participants may be more inclined to explore various strategies (i.e., hypothesis testing through frequent win-shift behavior) due to the heightened cognitive and physical effort required to navigate their bodies toward a goal. In contrast, button press tasks, which demand minimal physical or cognitive effort, might promote more conservative, win-stay behavior. While these results require further empirical testing, they do present compelling evidence that participants making decisions between options presented in a more natural setting may be computationally different from participants pressing buttons in simplistic lab-based experiments. Thus, the ability of current reinforcement learning models to predict behavior in simplistic lab-based experiments may be insufficient for explaining behavior and cognition in the complexity of naturalistic tasks and are ripe for future investigations. More generally, virtual reality and AR, both components of extended reality, enhance more realistic experimental conditions and reduce the inherent variability in manipulations aimed at replicating laboratory-controlled experimental outcomes in naturalistic scenarios. The adoption of these technologies allows for a nuanced examination of the interplay between internal validity—confidence in the causation inferred from the experimental design—and external/ecological validity, the extent to which results can be generalized to naturalistic settings (Loomis et al., 1999).

The observation of the reward positivity and adaptive behavior in this AR task strengthens the ecological validity (EV) of measures of goal-directed behavior. EV refers to three dimensions of experimentation (research setting, stimuli, and response) that should mimic the natural world as close as possible (Brunswik, 1943; Lewin, 1943; Schmuckler, 2001). Research setting EV concerns the environment in which the research takes place; stimuli EV addresses the issue of representativeness and naturalness of objects presented in an experiment; and response EV involves the nature of the task and behavior required from the participant (Brunswik, 1943; Schmuckler, 2001). The current study addresses all three EV dimensions of goal-directed behavior by (1) utilizing mobile-EEG and AR methods to create a realistic operant chamber (research setting EV), (2) demonstrating the ability to record ACC-related electrophysiological responses (e.g., the reward positivity) to holographic reward cues in the real world (stimuli EV), and (3) revealing adaptive responding based on positive and negative feedback (response EV). Regarding research setting EV, AR provides an opportunity to interweave and control experimental manipulations within the participants’ physical world, thereby altering their ongoing perception of events. While previous studies have used virtual reality environments to balance naturalistic observations and control (Campbell et al., 2009; Parsons, 2015), inherent limitations emerge—motion sickness, limited range of navigation, computer-generated environments that do not truly reflect “our world,” the inability to see their own bodies that creates a sense of disembodiment, and extensive training (Garrett et al., 2018). AR methods overcome these limitations, thereby providing an exciting opportunity to conduct future experiments beyond the laboratory and in more natural settings. In relation to stimuli EV, we found that reward-related holograms can elicit the reward positivity. As argued elsewhere, replication of neural signatures found in desktop tasks demonstrate the feasibility of such approaches while simultaneously accounting for EV of the task in general (Krugliak and Clarke, 2021). In one notable instance, Lange and Osinsky (2020) were able to replicate an increased frontal-midline theta responses to negative action outcomes during a naturalistic toy shooting task but failed to elicit feedback-related ERPs, likely due to the sensitivity of ERPs to latency jitter (Lange and Osinsky, 2020). Finally, regarding response EV, the AR task drove adaptive learning following negative feedback but failed to elicit a dominant win-stay strategy. Collectively, these findings underscore the promise of integrating mobile-EEG and AR to enhance the EV of reinforcement learning tasks (Krugliak and Clarke, 2021).

In sum, combining mobile-EEG with AR technology is a feasible solution to enhance the ecological validity of human electrophysiological studies of reinforcement learning and goal-directed behavior and holds promise for integrating experimental, computational, and theoretical analyses of goal-directed behavior in animals within the field of human mobile-EEG research. Future clinical applications for this paradigm could also uncover open questions of cue reactivity in drug addiction and other mental health disorders.

### Limitations

Although this research presents some of the first data using mobile-EEG and AR to examine electrophysiological and behavioral response properties during active goal-directed navigation, future research may address some of the study's limitations. First, while the ERP results were consistent with previous work, the amplitude of the reward positivity appeared smaller compared with the reward positivity recorded from stationary subjects in a highly controlled environments (Baker and Holroyd, 2009; Baker et al., 2016b). While undoubtfully active walking decreased the signal-to-noise ratio, and thus reduced the amplitude of the reward positivity and other feedback-related ERPs (e.g., P200), it is worth noting that the amplitude of other ERPs associated with sensation and perception (P100 and N170) were consistent with previous work (Baker and Holroyd, 2013). Future studies should attempt to dissociate whether the small amplitudes observed here was a result of methodological confounds (e.g., device-related trial-to-trial latency jitter may distort ERP amplitudes) or a natural phenomenon observed during complex naturalistic tasks (i.e., cognitive effort/energy may be distributed across multiple systems). Another limitation inherent to testing EV is that the more the environment is naturalistic, the less it lends itself to experimental control. Any natural phenomena existing during our day-to-day activity (e.g., weather, bystander interference) could affect the cognitive computations performed during a dynamic experiment. Since the present study was still conducted within the laboratory, the current AR task may not fully represent a true naturalistic setting. Hence, future experiments could test this paradigm in a more dynamic world full of distractions.

Further, while mobile-EEG is well suited for real-time recordings of naturalistic behavior due to its portability, low cost, and versatility, and can easily translate into therapeutic applications such as brain–computer interfaces (Nicolas-Alonso and Gomez-Gil, 2012), integrating AR/VR paradigms with other noninvasive imaging methods may also allow for neural recordings during movement, as demonstrated in studies using functional near-infrared spectroscopy (McKendrick et al., 2015; Mirelman et al., 2017; Pinti et al., 2020), and in stationary settings using optically pumped magnetometer-magnetoencephalography (Roberts et al., 2019). Future research could incorporate these methods to foster a more naturalistic and comprehensive approach to studying brain activity. Lastly, event-related oscillatory (ERO) responses may provide additional sources of information about neurocognitive processing underlying reinforcement learning (Yeung et al., 2004; Holroyd et al., 2012). For example, it is well known that unexpected, task-relevant events elicit a brief burst of power in the theta frequency range ∼200–300 ms after the event (frontal midline theta, FMT) that appears to index the deployment of control (Cavanagh and Frank, 2014). It has been previously proposed that these FMT activities could act to organize neural processes during decision points, such as where choice-relevant information is integrated to inform action selection (Cavanagh and Frank, 2014). Thus, both ERPs and ERO signals can provide both complementary and diverse sources of information during naturalistic reward tasks and should be considered in future studies. Finally, we did not test the EV of reinforcement learning models in this proof-of-concept study. Future studies should test whether conventional computational models of cognition can make quantitative predictions about observable behavior in both simplistic lab-based tasks and complex naturalistic tasks (Robles et al., 2021).

## Data Availability

The data can be provided by J.S.S. pending scientific review and a completed material transfer agreement. Requests for the data should be submitted to jss388@newark.rutgers.edu.

## Synthesis

Reviewing Editor: James Howard, Brandeis University Decisions are customarily a result of the Reviewing Editor and the peer reviewers coming together and discussing their recommendations until a consensus is reached. When revisions are invited, a fact-based synthesis statement explaining their decision and outlining what is needed to prepare a revision will be listed below. The following reviewer(s) agreed to reveal their identity: Nicholas Alexander, Clíona Kelly. Note: If this manuscript was transferred from JNeurosci and a decision was made to accept the manuscript without peer review, a brief statement to this effect will instead be what is listed below.

Your manuscript has been assessed by two reviewers. Both reviewers are enthusiastic about the use of a more naturalistic experimental approach to probe neural responses to reward prediction errors. Through a consultation process, we have come to the consensus that some concerns need to be addressed before this manuscript can be considered for publication. I will summarize these concerns below, with specific suggestions made by reviewers under each point:

1. One overarching concern relates to the way the findings are presented, and we have determined that some clarification of key points of the analysis methods is needed:

a. What was the basis for choosing the electrode positions you recorded? It seems like you would have benefited from recording frontal electrodes for the purpose of applying ICA to remove eye blink/saccade related artefacts. You would also have been able to capture frontal midline activity better. I suspect it is due to the hololens placement, but in my experience electrodes can sit comfortably underneath head mounted displays.

b. Related to the previous point about electrode positions, please clarify how you generated the topography shown in figure 3. It appears to show a gradient from frontal midline (blue) to frontal (red) which it was not possible to observe, even when extrapolating the signal. Please explain how the figure was produced. Generally the two options are to either use linear interpolation onto a grid (least assumptions) or to use the 'v4' interpretation method in Matlab which fits a surface to the data at each position. If it is unclear what BrainVision Analyzer is doing, perhaps try open analysis packages like Fieldtrip, SPM or Brainstorm.

c. For the ERP analysis, you report μV values which are generally system dependent, based on amplification and impedance values. You have enough subjects to run a group-level ERP analysis and report t-stats. Or you could report a relative measure like percent change. My background is more in event-related spectral perturbation analysis, in which people commonly use decibel conversion at the individual level and then apply stats to that at group level. In the limitations section you discuss small amplitudes observed and their possible source. Consider using a method like standardised measurement error to quantify this effect.

d. Line 122: Given the stimuli used, did you check if participants were colourblind?

e. Line 146: Was there any delay between the participant was standing in front of the orb and looking at it? Presumably the eyetracker had to wait some time to confirm they were fixated?

f. Were participants aware of the additional $5 they would gain if they completed the study? As the task reward is also focused on money, I'm considering the consequences this may have if they were aware of the additional $5. A sentence informing the reader of this would suffice.

g. Line 193: Did you measure electrode impedance? If you have it, please report the median value or the range.

h. Line 197: Why is this bandpass filter so tight? Were the data significantly contaminated by high frequency artefacts? What does the power spectrum look like before and after filtering? Considering you are looking at ERPs on the order of 2Hz, a 1Hz highpass filter may be causing some issues.

i. Line 199: How were eye blinks/saccades identified? Manually? How many components were removed? The dimensionality of your data is already limited - is ICA appropriate?

j. Line 207 Is there a reference for interpolating based specifically on 5% or more or is this an arbitrary number? If the latter, please explain why 5%. Please also include the average number of channels that were interpolated and/or if there were specific "problem" channels that were consistently interpolated for each participant. Similar to the comment above, why 5% and please include details on the number of trials excluded per condition, the average number of trials removed or another grouping of your choice.

k. Line 223: I have not come across this method for demonstrating the peak. Why do you need to employ any statistics at all? The topography will not change by running a t-stat. It is sufficient to say, the electrode with the greatest ERP peak X is at Y.

l. Line 243 Here you state participants completed 195 trials (which should read "on average 195 trials" if that is indeed correct) but on line 235-236, the two blocks add up to 200 trials and on 151 you state that there are four blocks and 50 trials per block. Please clarify this apparent discrepancy. Additionally, if some participants did not complete all the trials this should be clearly stated with reasons why and considerations that were taken during the analysis as a result of unequal trial numbers per block.

m. Line 251 It is unclear what "choice behavior" is referring to, as it has not been mentioned previously in the manuscript. If it refers to stay/shift behavior, please make reference to that in the Behavioral Analysis section where you explain win-stay and lose-shift behavior (line 231-233) by noting that this is categorized or later referred to as participants' choice behavior. Then use this term consistently when referring to stay/shift behaviors.

2. Another general concern surrounds the motivation to focus on analysis of ERPs rather than frontal midline theta oscillatory activity. The reviewers have suggested some optional additional analyses, but agree that at a minimum a clarification of the choice to focus on ERP's rather than oscillations is necessary:

a. You discuss frontal midline theta in the introduction and in the discussion. Oscillatory signals are perhaps more amenable to analysis in naturalistic tasks as they are less reliant on time-locking. Have you looked at whether a time-frequency analysis of your data contains expected frontal midline theta activity? The ERP analysis is sufficient, but you may get more out of the data this way. The results may also generalise to the kinds of reward signals that are likely to be observable in the real world.

3. There are some additional suggestions regarding wording and the organization of some parts of the manuscript that should be addressed:

a. The claim in the title that signals are being measured in the 'real world' is a bit strong. The behaviours exhibited in the task are still quite unnatural, despite the freedom of movement and naturalistic stimuli used. I suggest changing the title to something like "Recording neural reward signals from a naturalistic task using mobile-EEG and augmented reality". An additional suggestion along these lines for line 25 is to stick to a general definition of headset-based AR, which is to superimpose virtual images onto the real-world view. Generally, I would recommend readdressing discussions in the paper about 'real-world' versus controlled lab experiments. This was still a highly contrived experience for participants. Focus on the real differences between your task and computerised button press tasks.

b. I suggest avoiding the term hologram when talking about headset based augmented reality. They are not really holograms. You would be better off describing what the virtual object is. For example, "the virtual sign". Or, "The set-up consisted of pacing three virtual objects within the room: the start portal..."

c. In the Introduction section you've included information about the technique used and the aim to replicate lab-based experiments, but there isn't an explicit sentence on how you will do that, i.e. by asking participants to physically walk towards prespecified locations. Near the end of your intro please include a short paragraph or a couple of sentences that give a brief overview of what the task is, so the reader is set up to understand the methods e.g. "In this paradigm, we aimed to investigate...by asking participants to..."

d. Break up the final paragraph of the Introduction, so that the first paragraph discusses the benefits of VR/AR

e. In the Methods section, please clarify whether you collected any information on whether participants have experience with AR/HoloLens. Additionally, please describe what, if any, training the participants were provided with the system before the experiment began.

f. In the Methods section, please clarify whether participants were given cues or clues as to which location may or may not have a reward, or if they were instructed to choose randomly. Also include a description of

whether each trial was a forced choice or not, i.e. they cannot progress to the next trial until they complete the current one or, they X seconds to begin moving/to get to the target before the next trial would begin.

g. In your discussion it would be worth mentioning why EEG is suitable for this kind of research. What is the state of the field for other imaging modalities and naturalistic imaging? What about fNIRS and OPM-MEG?

h. If you have any suggestions as to why you think there was a <200ms delay, please include that in your discussion or limitations.

i. Line 27: It is not necessary to report age in the abstract.

j. Line 38: You do not need to emphasise that this is the first study to combine EEG and AR to investigate goal-directed behaviour. That is quite a niche claim and the distinction between AR/VR and general naturalistic stimuli is quite blurred in terms of its application to neuroscience.

k. Line 115: Remove "and all experiments were performed in accordance with relevant guidelines and regulations". That is too general.

l. Line 140 "To begin, participants calibrated their position within the room by placing a human icon hologram over a floor sticker..." Mention the reason for placing these items around the room.

m. Lines 145-146 This is the first time we're being told about the participant's task, make it more clear. At the moment you have written the behavior that they performed but the reader needs to know the question or task they were told/given beforehand as the task in question may elicit different cognitive responses e.g. "Participants' were tasked with choosing one of the goal locations (left, right)..."

n. Line 170 "1=event, 0=nonevent) for each participant (Fig. 1A)". This does not make sense to me. Could this section be rephrased to reflect the audience reading the article?

o. Line 171: hz to Hz.

p. Line 175 Assuming the delay was consistent "there was a consistent but unexpected delay..."

q. Line 179: Is this detail about the trigger timing necessary? If you share the data with someone they will need to know, but it does not affect the article content as far as I can see.

r. Line 202: Change to "and a difference wave representing reward positivity (REWP, black)" or similar.

s. Line 237: The information about exclusions is general, not just for behaviour data. Include it in the participant info section at the start of methods.

4. Suggestions to add references to your manuscript or clarify some references to better contextualize your experiment and findings:

a. There is an opportunity in the introduction to relate the work presented in your article to the general movement towards naturalistic imaging/stimuli/behaviour in neuroscience. Of particular relevance here is the work of MoBi groups, such as Gramann et al (2014), "Imaging natural cognition in action". You could also look at the Sonkusare, Breakspear, Guo (2019) or Stangl, Maoz, Suthana (2023). For balance, see Rust &Movshon (2005).

b. Line 61: Translational neuroscience has contributed a great deal to this area as well. See Haber &Knutson, Neuropsychopharmacology, 2010.

c. Line 88: Please cite a relevant source on FM theta. Cavanagh and Frank (2014), for example.

d. Line 103: Loomis, Blascovich and Beall (1999) presented these ideas about how VR (incorporating AR/XR in this case) can reduce the relationship between internal validity and external validity.

e. Line 129: Did you use any plugins/toolboxes for Unity? If so you do not need to cite all of them, but if you used some of the tools available for making experiments (i.e. work by Jack Brookes or BMLtux) then you should mention it.

f. Line 193: Which is Lin et al., 2022 cited here?

5. Suggestions for changes to figures:

a. Can you export your figures in higher DPI?

b. Line 139 Rings in Figure 1A are purple and the counter is blue/aqua - This may be a personal perception of colors but consider changing the colors to something less ambiguous. Also, refer to the Figure again at this point. Alternatively, I think using a blue shape similar to the hologram instead of the rings would be better (this way it will match what is seen in the video which is very informative for this section).

c. Figure 1A, Rightward trajectory path length is missing. If the return value written is for both left and right trajectories, make that clear. In the corners where the green tick or red cross is, include both images with an "or" in between to make it clear that one corner isn't always right/wrong. Please reiterate that the three ascending rings denote the start portal.

d. Figure 1C, It is a little counterintuitive as we read from left to right - it would make sense for the timeline to be flipped on the y-axis or include an arrow on the x-axis with the label "time" to denote the direction of it.

e. Figure 2B, Similar to Figure 1, Section C, reading from left to right, RTs should be on the left hand side of the graph. The horizontal bar in between Return and Stay isn't helpful, visually. If its purpose is to help separate the x values, maybe add a very short horizontal bar between the values themselves.
